# m^6^A mRNA Methylation Regulates LKB1 to Promote Autophagy of Hepatoblastoma Cells through Upregulated Phosphorylation of AMPK

**DOI:** 10.3390/genes12111747

**Published:** 2021-10-30

**Authors:** Guohui Li, Liang Deng, Nan Huang, Zhongqi Cui, Qi Wu, Ji Ma, Qiuhui Pan, Fenyong Sun

**Affiliations:** 1 School of Life Sciences, Jiangsu University, Zhenjiang 212013, China; ghli@ujs.edu.cn (G.L.); denglianghp@163.com (L.D.); 2 Department of Clinical Laboratory Medicine, Shanghai Tenth People’s Hospital of Tongji University, Shanghai 200072, China; huang_nan2021@163.com (N.H.); 18817527896@163.com (Z.C.); wuqi496@163.com (Q.W.); 3 Department of Laboratory Medicine, Shanghai Children’s Medical Center, Shanghai Jiao Tong University School of Medicine, Shanghai 200072, China; maji@scmc.com.cn (J.M.); panqiuhui_med@163.com (Q.P.); 4 Shanghai Key Laboratory of Clinical Molecular Diagnostics for Pediatrics, Shanghai 200072, China

**Keywords:** WTAP, HCC, autophagy, LKB1, AMPK

## Abstract

The N6-methyladenosine (m^6^A) RNA modification can regulate autophagy to modulate the growth and development of tumors, but the mechanism of m6A modification for the regulation of autophagy in hepatocellular carcinoma cells (HCC) remains unclear. In the study, the knockdown of the Wilms’ tumor 1-associating protein (WTAP) was made in HCC to study the correlation between m6A modification and autophagy. A fluorescent confocal microscopy analysis showed that the knockdown of WTAP could facilitate the autophagy of HCC. A Western blot analysis showed that the level of p-AMPK was decreased in WTAP-knockdown HCC cells. Additionally, LKB1, the upstream kinase of AMPK, was regulated by WTAP and it could mediate the phosphorylation of AMPK in an m6A-dependent manner. Further studies revealed that the knockdown of WTAP could reduce the level of LKB1 mRNA with m6A. This could result in the increased stability of LKB1 mRNA to promote its expression. The knockdown of WTAP could upregulate the level of autophagy and inhibit HCC proliferation. However, the overexpression of WTAP could resist autophagic cell death.

## 1. Introduction

Liver cancer is a frequently occurring malignancy, and it ranks among the highest in the world in terms of cancer-related death. The incidence of liver cancer in China is ranked first in the world, and the situation of liver cancer prevention and treatment is still very serious [1,2]. So, it is very important to develop a more effective treatment for patients of liver cancer. Autophagy is an evolutionarily conserved intracellular degradation and metabolism process and it plays important roles in maintaining cell metabolism, genome integrity, and the self-renewal of organelles [3,4]. Meanwhile, autophagy is also a double-edged sword in tumor cells. The inhibition of autophagy can increase the sensitivity of cancer cells to anti-cancer therapy, but excessive autophagy can cause autophagic cell death. Therefore, autophagy may be a potential therapeutic target in the treatment of tumors. The cellular process of autophagy involves a series of steps including the expansion of an isolation membrane, sequestration, autophagosome formation and lysosomal degradation of cytosolic components such as damaged organelles and misfolded proteins [5]. Autophagy is a highly regulated biological process mediated by various proteins to maintain cellular function. To date, more than 40 autophage-related genes have been identified to be involved with the formation of autophagosome, and at least 15 genes are involved in regulation of autophagy in yeast undergoing nutrient deprivation [6,7]. However, the abnormal regulation of autophagy can cause the pathogenesis of numerous human diseases, including cancers [8,9].

Recently, much attention has focused on the N-6-methyladenosine (m^6^A) modification, one of the most abundant post-transcriptional modifications, and this modification is reported to be involved in a variety of biological processes [10,11,12]. The functional components of the RNA methyltransferase complex includes methyltransferase-like 3 (METTL3), methyltransferase-like 14 (METTL14), and Wilms tumor 1-associated protein (WTAP) [13,14]. Increasing evidence shows that the m^6^A of RNA modification is involved in a variety of cellular events including mRNA stability, mRNA decay, translation, alternative splicing, and subcellular localization [15,16,17]. The m^6^A level can be regulated by some cellular factors. For example, Yang et al. reported that miR-145 could modulate m6A levels by targeting the 3′-UTR of YTHDF2 mRNA in HCC cells [18]. Therefore, the dysregulation of m^6^A is closely related to the occurrence and development of human tumors. For example, Ma et al. reported that METTL14 could suppress the metastatic potential of hepatocellular carcinoma by modulating the m^6^A of RNA [19]. Liu et al. reported that the dysregulated N6-methyladenosine methylation writer METTL3 contributed to the proliferation and migration of gastric cancer [20]. Han et al. summarized that the dysregulation of m^6^A-containing gene expression via the abnormal expression of m^6^A methyltransferases, demethylases, or reader proteins were closely associated with tumorigenicity [21]. Therefore, m^6^A modification can dynamically regulate gene expression in response to a variety of physiologic and pathologic stimuli. To date, some autophagy-related genes are reported to be regulated by m^6^A modification. For example, the autophagy protein ULK1 was positively correlated with the demethylase FTO, ant it could act on the ULK1 transcript, regulating its protein level and stability [22]. Wang et al. reported that m^6^A mRNA methylation controlled autophagy and adipogenesis by targeting Atg5 and Atg7 [23]. However, the regulatory mechanism of m^6^A on autophagy remains unclear.

In this study, the increased abundance LKB1 mRNA was correlated with the reduced level of m^6^A in WTAP-knockdown HCC. Furthermore, the upregulated LKB1 could increase the phosphorylation of AMPK, which could facilitate the level of autophagy in HCC. 

## 2. Materials and Methods

### 2.1. Cell Culture

HEK-293T was purchased from the American Type Culture Collection (Manassas, VA, USA), and the HEK-293T cells was used to propagate Lentiviruses HEK-293T and SMMC-7721 cells were cultured in Dulbecco’s modified Eagle’ medium (Gibco, Carlsbad, CA, USA). HL-7702, BEL-7402, and BEL-7404 cells were cultured in RPMI 1640 medium (Gibco). All media are supplemented with 10% fetal bovine serum (Gibco) and 1% penicillin/streptomycin (Gibco). All cells were cultured at 37 °C and 5% CO_2_.

### 2.2. Viruses, Plasmids and Transfection

Some lentiviruses were used for WTAP knockdown, and the overexpression of WTAP fusion with EGFP. shWTAP-1 (target sequence: GCAAGAGUGUACUACUCAA) or shWTAP-2 (target sequence: GCCCAACUGAGAUCAACAA) used for control were obtained from GenePharma (Suzhou, China). After HCC cells were infected with corresponding shRNA, 2 μg/mL puromycin (Sigma Aldrich, Gillingham, UK) was used to screen and achieve stable cell lines. Small interfering RNA (siRNA: 5′-CGUGUGUAUGAACGGCACA-3′) against LKB1 were synthesized by GenePharma. Lipofectamine 2000 kit (Invitrogen, Carlsbad, CA, USA) was used for transient transfection with siRNA according to the manufacturer’s instructions. 

### 2.3. RNA Extraction and Quantitative PCR (qPCR) Analysis

Trizol^®^ reagent (Invitrogen, Carlsbad, CA, USA) was used to extract total RNA from cells according to the manufacturer’s instructions. Briefly, cells were lysed with TRIzol reagent at room temperature for more than 5 min and chloroform was added. After centrifugation at 12,000× *g* rpm for 15 min, the aqueous phase was collected and mixed with isopropanol before centrifuging at 12,000× *g* rpm for 10 min at 4 °C. RNA was dissolved in RNase-free water and recovered by centrifuge. cDNA was synthesized using PrimeScriptTM RT reagent Kit (TaKaRa, Kusatsu, Japan). The resulting cDNA was used as template to perform RT-qPCR on ABI 7900HT PCR sequencer (Applied Biosystems, Waltham, MA, USA) with TB Green^®^ Premix Ex Taq™ II (TaKaRa). qWTAP-F: 5′-CTTCCCAAGAAGGTTCGATTGA-3′ and qWTAP-R: 5′-TCAGACTCTCTTAGGCCAGTTAC-3′ were designed to determine the abundance of WTAP transcript through RT-qPCR. Primers 5′-TGCACCACCAACTGCTTAGC-3′ and 5′-GGCATGGACTGTGGTCATGAG-3′ were used to amplify the internal control of GAPDH. 

### 2.4. High-through Sequencing and Bioinformatic Analysis

To define the targets of m^6^A affecting the level of autophagy, two groups of shCON and shWTAP-treated cells were collected. Total RNAs were extracted from the cells using Trizol (Invitrogen, Carlsbad, CA, USA), which were used for high-throughput sequencing (Oebiotech Corporation, Shanghai, China). Bioinformatics analysis was made to identify the differential expression of some genes through Go analysis and KEGG pathway analysis.

### 2.5. MeRIP-qPCR Analysis

Fragmented mRNA with m^6^A modification enrichment was performed using Magna MeRIP m^6^A Kit (Millipore, Burlington, MA, USA), which was followed by RT-qPCR to determine the m^6^A level of target genes according to the manufacturer’s instructions. Briefly, 5 μg of fragmented mRNA was incubated with 5 μg m^6^A antibody (202, 003, Synaptic Systems) or mouse IgG-conjugated beads (CS200621, Millipore, Boston, MA, USA) in 500 μL 1× IP buffer for 4 h at 4 °C. Methylated RNA was eluted by free m^6^A from the beads and purified with RNeasy Minikit (217, 004, Qiagen). One tenth of the fragmented RNA was saved as an input control for standardization. The relevant enrichment of LKB1 mRNA with m^6^A modification in each sample was analyzed by RT-qPCR using qLKB1-F: 5′-TCTACAACATCACCACGGGTC-3′ and qLKB1-R: 5′-TTCGTACTCAAGCATCCCTTTC-3′.

### 2.6. Western Blot Analysis

RIPA buffer containing phosphatase inhibitor and protease inhibitor (Sigma Aldrich) was used to lyse the cells. Protein concentrations of cell lysates were determined with BCA Protein Assay kit (Pierce) according to the manufacturer’s instructions. Total protein (30 μg) in each sample was separated by SDS-PAGE gel and transferred to nitrocellulose membranes. The membranes were blocked with 5% bovine serum albumin (BSA) for one hour at room temperature. Then, incubation was made with primary antibodies against WTAP (ab195380, Abcam, Cambridge, MA, USA), GAPDH (ab128915, Abcam, Cambridge, MA, USA), AMPK (# 4150S, Cell Signaling Technology, CST, Danvers, MA, USA), p-AMPK (# 50081S, CST), ULK1 (# 8054S, CST), p-ULK1 (# 6888S, CST), Beclin 1 (# 3738S, CST), mTOR (# 2972S, CST), p-mTOR (S2448) (#2971S, CST), p-mTOR (S2481) (# 2974S, CST), LC3I/II (# 4108S, CST), and LKB1 (A2122, Abclone, Wuhan, China) at 4 °C overnight. Subsequently, the membranes were incubated with fluorescently labeled goat anti-mouse IgG (926-32210, LICOR) or goat anti-rabbit IgG (926-32211,LICOR) secondary antibodies for one hour at room temperature, respectively. Finally, the fluorescence hybridization signal was detected by the Odyssey system (LI-COR, Lincoln, NE, USA). 

### 2.7. Confocal Microscopy

The indicated cells were fixed with 4% paraformaldehyde (Sigma Aldrich) for 15 min at room temperature, and then cells were permeabilized with 0.5% Triton X-100 for 20 min at 37 °C. After blocking with 5% BSA in PBS for 1 h at room temperature, cells were incubated with the indicated antibodies. Nuclei were stained with 4′, 6-diamidino-2-phenylindole (DAPI, Sigma Aldrich). Finally, the images of fluorescence were acquired with the confocal laser-scanning microscope (Carl Zeiss, Jena, Germany).

### 2.8. RNA Stability Assays

To determine the life-time of LKB1, actinomycin (5 mg/mL) was used to incubate with the cells for transcriptional termination. Samples were collected at 0, 0.5, 4, 8 h post-termination, respectively. The total RNA was extracted and the remaining LKB1 transcripts was determined by RT-qPCR.

### 2.9. Cell Viability Assay

SMMC-7721 WTAP-OE and SMMC-7721 CON-OE cells (1 × 10^3^ cells/well) were seeded into 96-well plates and incubated at 37 °C in a humidified 5% CO_2_ atmosphere overnight, respectively. The cells were continuously cultured for 1 to 6 days in vitro. Cell Counting Kit-8 (CCK8, Beyotime, Haimen, China) was used to treat the cells at indicated time point. The optical density at 450 nm (OD450) was measured using a multifunctional microplate reader (BioTek, Winooski, VT, USA). Cell viability was expressed as a percentage of the control.

### 2.10. Colony Formation Assay

SMMC-7721 WTAP-OE and SMMC-7721 CON-OE cells (500 cells/well) were seeded into 12-well plates and continuously cultured for 14 days. The colonies were fixed in absolute ethanol for 15 min, stained with 0.1% crystal violet solution for 30 min, and then images were collected.

### 2.11. Statistical Analysis

All data were shown as mean ± standard deviation (SD) from three independent experiments. Statistical analysis was performed using SPSSv22.0 (IBM, Armonk, NY, USA). Differences were analyzed by Student’s-test for two groups or one-way ANOVA for multiple groups. All experiments were performed three times and the *p* values < 0.05 were considered statistically significant.

## 3. Results

### 3.1. Knockdown of WTAP Facilitating Autophagy

**Figure 1:**
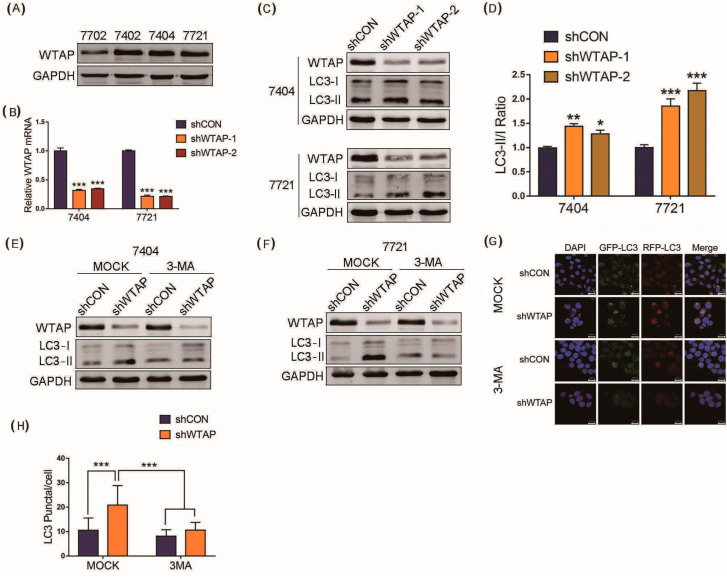
Knockdown of WTAP facilitating autophagy of HCC. (**A**) Western blot analysis of basal expression of WTAP in different HCC cell lines. (**B**) RT-qPCR analysis of WTAP transcripts from shWTAP cells and shCON cells. (**C**) Western blot analysis of LC3-I and LC3-II in shWTAP SMMC-7721 cells and shWTAP BEL-7404 cells. (**D**) Statistical analysis of LC3 II/LC3 I ratio between shCON and shWTAP group. (**E**) Western blot analysis of LC3-I and LC3-II in WTAP-knockdown BEL-7404 cells treated with or without 3-MA. (**F**) Western blot analysis of LC3-I and LC3-II in WTAP-knockdown SMMC-7721 cells treated with or without 3-MA. (**G**) Cofocal microscopy analysis of LC3 in shWTAP cells treated with or without 3-MA. (**H**) Statistical analysis of LC3 puncta in shWTAP cells and shCON cells, as well as 3-MA-treated cells. Error bars indicate the mean ± SD from three independent experiments (*n* = 3). *p* values was used to indicate statistica difference. * *p* < 0.05, ** *p* < 0.01 and *** *p* < 0.001.

Western blot analysis was first performed to examine the basal expression level of WTAP in several HCC cell lines (BEL-7402, BEL-7404 and SMMC-7721) and the normal hepatic cell line (HL-7702). The results indicated that the expression level of WTAP was higher in the HCC cell lines than that in the normal hepatic cell line (Figure 1A). Then, BEL-7404 and SMMC-7721 cell lines were selected for the study in view of the high expression of WTAP. Lentiviruses encoding short hairpin RNA (shWTAP-1, or shWTAP-2) were propagated in HEK-293T, which was used to decrease the expression of WTAP in the BEL-7404 and SMMC-7721 cell lines, respectively. The RT-qPCR results indicated that the abundance of WTAP transcripts decreased sharply in shWTAP-1 or shWTAP-2 cells, and there was significant difference between the shWTAP cells and control groups (Figure 1B). Furthermore, the Western blot results showed that the expression level of WTAP in shWTAP cells was obviously lower than that in the control group (Figure 1C). As we know, LC3-phospholipid conjugate (LC3-II) is localized on autophagosomes and autolysosomes, and the conversion of LC3-I into LC3-II is indicative of autophagosome formation. Therefore, the LC3-II/LC3-I ratio is widely used as a marker for autophagy. In the study, the knockdown of WTAP significantly increased the LC3-II/LC3-I ratio compared with that of control group (Figure 1C), and the statistical analysis of LC3 II/LC3 I ratio showed a significant difference between the shCON and shWTAP cells (Figure 1D). 

LC3, fused with the green fluorescent protein (GFP) or red fluorescent protein (RFP), were further used to study the production of the autophagosomes in shWTAP cells and shCON cells through the observation of the fluorescence signal. Furthermore, autophagy inhibitors were used to scrutinize whether the knockdown of WTAP affected the autophagy level of HCC in shCON and shWTAP cells treated with 3-methyladenine (3-MA). The Western blot results indicated that the knockdown of WTAP could facilitate the production of autophagosomes compared with that in shCON group, but the upregulated level of autophagosomes by shWTAP could be significantly inhibited by 3-MA (Figure 1E,F). The fluorescence signal observation confirmed the increased number of LC3 spots in shWTAP cells. However, the number of LC3 spots were also decreased after the treatment with autophagy inhibitor 3-MA (Figure 1G). Furthermore, a statistical analysis showed that there was significant difference in LC3 puncta between the shWTAP cells and shCON cells, as well as the shWTAP cells and 3-MA-treated cells (Figure 1H), Taken together, the above results indicated that the knockdown of WTAP could facilitate the increased level of autophagy in HCC cells. Therefore, the upregulation of autophagy by the inhibitory expression of WTAP may be an effective strategy to inhibit the growth and metastasis of HCC.

### 3.2. AMPK Pathway Involved in Autophagy

**Figure 2:**
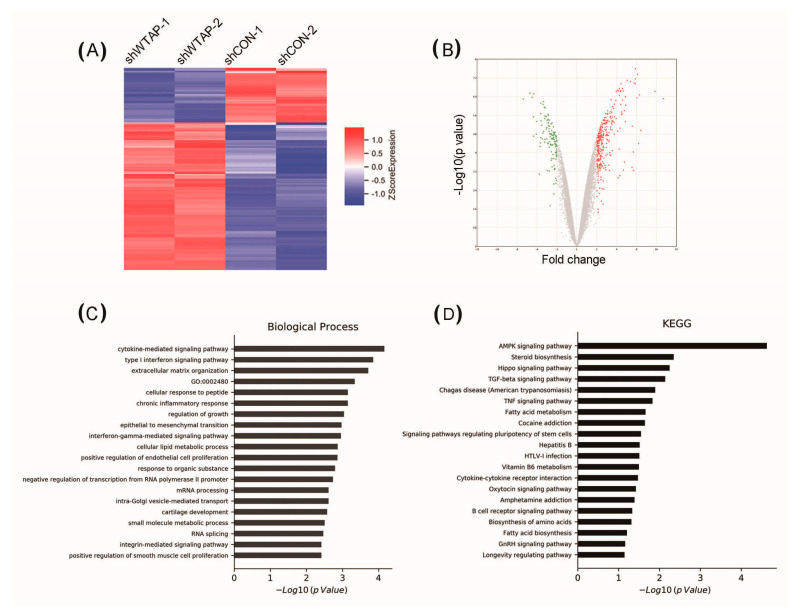
Analysis of differential expression of genes in shWTAP SMMC-7721 cell lines by high-throughput sequencing. (**A**) Heat map showing the differentially expressed genes between shCON and shWTAP SMMC-7721 cells. (**B**) Volcano map showing the differentially expressed genes between shCON and shWTAP SMMC-7721 cells. (**C**) GO analysis of differentially expressed genes involved with biological process between shCON and shWTAP SMMC-7721 cells. (**D**) KEGG signal pathway analysis showing differentially expressed genes between shCON and shWTAP SMMC-7721 cells.

The knockdown of WTAP in HCC cells resulted in increased of levels of autophagy. However, it is not clear that the regulatory mechanism of WTAP affects the autophagy levels of HCC. Meanwhile, it is also unknown whether some signaling pathways are involved in the regulation of autophagy levels. Therefore, some differential expression proteins between SMMC-7721 shCON and SMMC-7721 shWTAP cell lines were screened by high-throughput sequencing. A heat map showed that differential expressions of genes existed in the SMMC-7721 shCON and SMMC-7721 shWTAP cell lines (Figure 2A). Additionally, a volcano map also showed the differential expression of genes after the knockdown of WTAP in HCC cells (Figure 2B). 

Subsequently, the Gene Ontology (GO) database and the Kyoto Encyclopedia of Genes and Genomes (KEGG) database were used for an enrichment analysis of these differential genes. The results indicated that the differential genes were mainly involved in energy metabolism through GO analysis (Figure 2C). Additionally, a KEGG analysis further revealed differential genes mainly involved in the regulation of the AMPK signaling pathway after the knockdown of WTAP in HCC (Figure 2D). At present, the AMPK signaling pathway is reported to participate in the regulation of cellular autophagy [24,25]. Therefore, the knockdown of WTAP could facilitate the AMPK signaling pathway of HCC, which may be responsible for the regulation of intracellular autophagy.

### 3.3. Autophagy Facilitated by Upregulated Phosphorylation of AMPK

**Figure 3:**
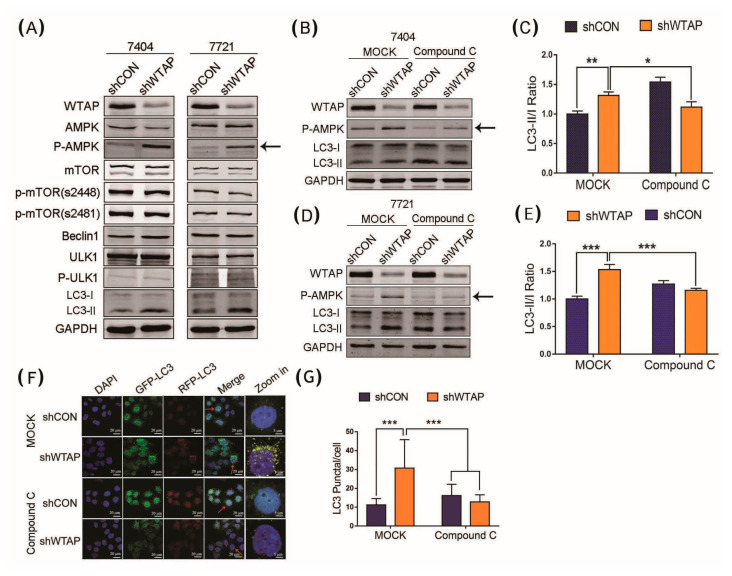
Effect of the upregulated level of p-AMPK caused by shWTAP on HCC autophagy. (**A**) Western blot analysis of some autophagy-related proteins expressed in BEL-7404 and SMMC-7721 cells. (**B**) Western blot analysis of LC3-I and LC3-II in shWTAP BEL-7404 cells treated with or without Compound C. (**C**) Western blot analysis of LC3-I and LC3-II in shWTAP BEL-7721 cells treated with or without Compound C. (**D**) Statistical analysis of LC3 II/LC3 I ratio between shCON and shWTAP–treated 7404 cell lines. (**E**) Statistical analysis of LC3 II/LC3 I ratio between shCON and shWTAP-treated 7721 cell lines. (**F**) Confocal fluorescence image of shWTAP cells treated with or without Compound C. (**G**) Statistical analysis of the numbers of LC3 puncta/cell. The cells indicated with red arrows were enlarged on the right. Error bars indicate the mean ± SD from three independent experiments (*n* = 3). * indicate a statistically significant difference (*p* < 0.05), ** indicate a statistically significant difference (*p* < 0.01) and *** indicate a statistically significant difference (*p* < 0.001).

To further demonstrate how the knockdown of WTAP facilitated the autophagy level of HCC, a Western blot was performed to examine the expression level of some of the autophagy-related proteins in shWTAP HCC cells. The results showed that the expression levels of some autophagy-related proteins, including Beclin1, ULK, p-ULK, mTOR, and p-mTOR did not vary significantly in the stable strains of shWTAP BEL-7404 and shWTAP SMMC-7721 cell lines. However, the phosphorylated AMPK and p-AMPK, as an autophagy-related proteins in the shWTAP BEL-7404 and shWTAP SMMC-7721 cell lines, were found to be significantly upregulated compared with that of the control group (Figure 3A). To further demonstrate the mechanism by which WTAP regulated the abundance of p-AMPK to facilitate the autophagy level in HCC, the AMPK phosphorylation inhibitor of Compound C was used to study the correlation between p-AMPK and autophagy. After the replacement with a complete medium containing Compound C (10 μM) in shCON and shWTAP cells for 2 h, the Western blot results showed that Compound C could significantly inhibit the increase in the autophagy level in the shWTAP cells, according to the LC3-II/LC3-I ratio (Figure 3B,D). A statistical analysis showed that there was significant difference in the LC3 II/LC3 I ratio between shCON and shWTAP cells (Figure 3C,E).

The above results indicated that the upregulated phosphorylation of AMPK was directly correlated with the enhancement of autophagy in shWTAP cells. Furthermore, confocal fluorescence microscopy showed that the number of LC3 spots was significantly increased in shWTAP cells compared with that in shCON cells, but the AMPK phosphorylation inhibitor of Compound C could decrease the number of LC3 spots in shWTAP cells (Figure 3F). Statistical analyses indicated that there was significant difference in the LC3 puncta between shCON and shWTAP cells, as well as shWTAP cells and Compound C-treated shWTAP cells (Figure 3G). Therefore, it was regarded that the knockdown of WTAP in HCC could facilitate the cellular autophagy level through the upregulation of p-AMPK.

### 3.4. Knockdown of WTAP Enhancing the Stability of LKB1 mRNA

**Figure 4:**
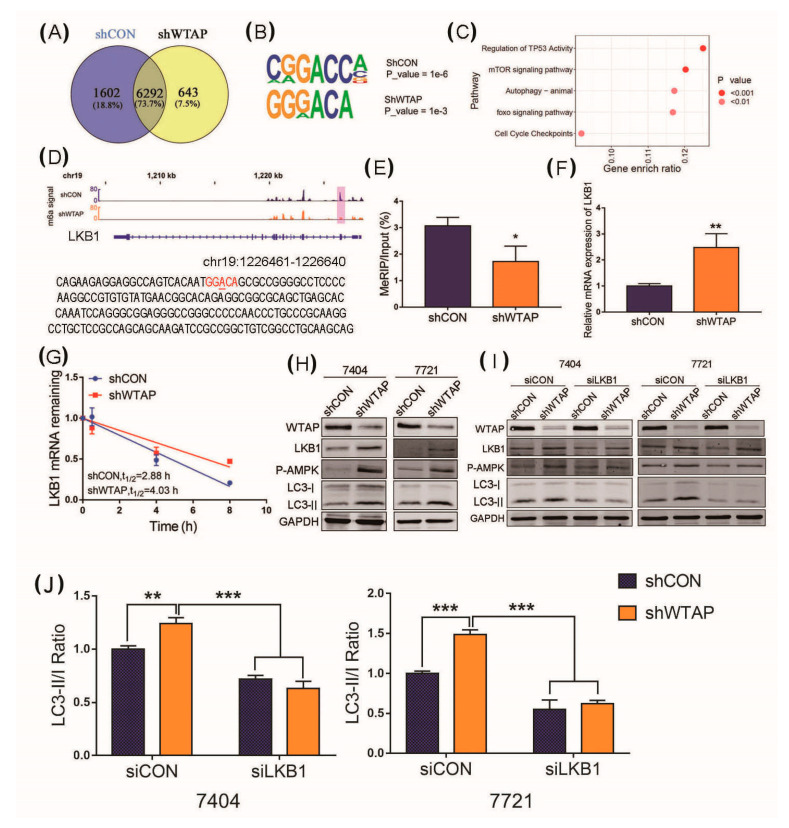
Knockdown of WTAP facilitating autophagy through upregulation of LKB1. (**A**) Venn diagram showing the difference in m^6^A peaks between shCON and shWTAP cell lines. (**B**) Conserved m^6^A motif found in schON and shWTAP HCC cell lines. (**C**) Signal pathway analysis of the different m^6^A modified genes between shCON and shWTAP HCC cell lines. (**D**) The m^6^A motif found in LKB1 transcript through sequencing. (**E**) MeRIP-qPCR analysis of cellular m^6^A levels in shCON and shWTAP HCC cell lines. (**F**) RT-qPCR used for the abundance analysis of LKB1 transcript in shCON and shWTAP HCC cell lines. (**G**) Half-life analysis of RNA stability of LKB1 transcript in shCON and shWTAP HCC cell lines. (**H**) Western blot analysis of LKB1, p-AMPK, LC3-I and LC3-II in shCON and shWTAP cells. (**I**) Western blot analysis of LKB1, p-AMPK, LC3-I and LC3-II in shCON and shWTAP cells, as well as in siLKB1 cells. (**J**) Statistical analysis of LC3 -II/LC3-I ratio between shCON and shWTAP-treated 7404 and 7721 cell lines. Error bars indicate the mean ± SD from three independent experiments (*n* = 3). * indicate a statistically significant difference (*p* < 0.05) and ** indicate a statistically significant difference (*p* < 0.01), *** indicate a statistically significant difference (*p* < 0.001).

The above results indicated that the abundance of p-AMPK was increased in shWTAP cell lines and could facilitate the autophagy level. However, the mechanism of the phosphorylation level of AMPK, regulated by the m^6^A modification, remains unclear. Liver kinase B1 (LKB1), an upstream protein of AMPK, was known as a serine/threonine kinase [26], which was regarded to play a critical role in the regulation of the phosphorylation level of AMPK. Therefore, LKB1 was selected to further study the correlation between the increased level of p-AMPK and m^6^A modification in shWTAP cell lines. We first compared the m^6^A peaks between the shCON and shWTAP cell lines. The result indicated that 2245 different m^6^A peaks were found between the shCON and shWTAP cell lines (Figure 4A). As we know, most m^6^A sites were found in conserved motifs and some conserved m^6^A motifs were also found in these different m^6^A peaks (Figure 4B), which was in line with the consensus reached by the RRACH sequence (where R represents a purine, A is m^6^A, and H is a nonguanine base).

Subsequently, an enrichment analysis of the differential genes with an m^6^A modification in shCON and shWTAP cell lines was conducted. The signal pathway analysis showed that these differential genes had a significant positive correlation with autophagy (Figure 4C). To further reveal the mechanism of the knockdown of WTAP, which facilitates AMPK phosphorylation, the transcript abundance and stability of LKB1 mRNA were analyzed in the study. The sites of the m^6^A modification in the LKB1, the upstream kinase of the AMPK, transcript was analyzed. The methylated RNA immunoprecipitation quantitative PCR (MeRIP-qPCR) was performed to examine the abundance of LKB1 transcripts from shCON and shWTAP cell lines. The results indicated that the conserved m^6^A modification site of GGACA could be found in the LKB1 mRNA (Figure 4D). Moreover, the MeRIP-qPCR results showed that the m^6^A modification level of LKB1 mRNA in the shWTAP cell line was decreased compared with that in the shCON cells (Figure 4E). However, the abundance of LKB1 mRNA was increased compared with that in shCON cells (Figure 4F). These results indicated that the knockdown of WTAP decreased the m^6^A level in LKB1 mRNA, but the abundance of LKB1 mRNA in shWTAP cells was increased. After LKB1 mRNA was extracted from shWTAP cells and shCON cells treated with Actinomycin (ActD), respectively, RT-qPCR was used to examine the stability of LKB1 mRNA. An analysis of the mRNA stability showed that the knockdown of WTAP could prolong the half-life of LKB1 mRNA transcripts, compared to the half-life of shCON cells (Figure 4G). Therefore, it was regarded that the expression level of LKB1 mRNA in shWATP cells was increased by the enhancement of LKB1 mRNA stability.

Subsequently, a Western blot analysis was used to examine the effect of shWTAP on the expression level of LKB1, p-AMPK, LC3-I, and LC3-II. The results showed that the LKB1 protein and p-AMPK levels were significantly increased in shWTAP BEL-7404 and shWTAP SMMC-7721 cells compared with those in shCON cells (Figure 4H). Therefore, it was regarded that the knockdown of WTAP could facilitate the phosphorylation level of AMPK through the upregulation of LKB1. However, it is unclear whether the increased level of LKB1 caused by shWTAP is directly correlated with the upregulation of autophagy. To clarify the question, LKB1 was knocked down in the shWTAP stable strain, and Western blot was further used to examine the expression of p-AMPK, LC3-I, and LC3-II for the evaluation of autophagy in HCC. The results showed that the knockdown of LKB1 could inhibit the phosphorylation of AMPK and LC3-II (Figure 4I). Furthermore, a statistical analysis of the LC3-II/LC3-I ratio was performed according to the expression of LC3-II and LC3-I, as shown in Figure 4I, and the comparison of the LC3-II/LC3-I ratio showed a decreased level of autophagosomes in siLKB1 cells compared with those in the siCON group (Figure 4J).

### 3.5. Overexpression of WTAP Facilitating Cell Proliferation

**Figure 5:**
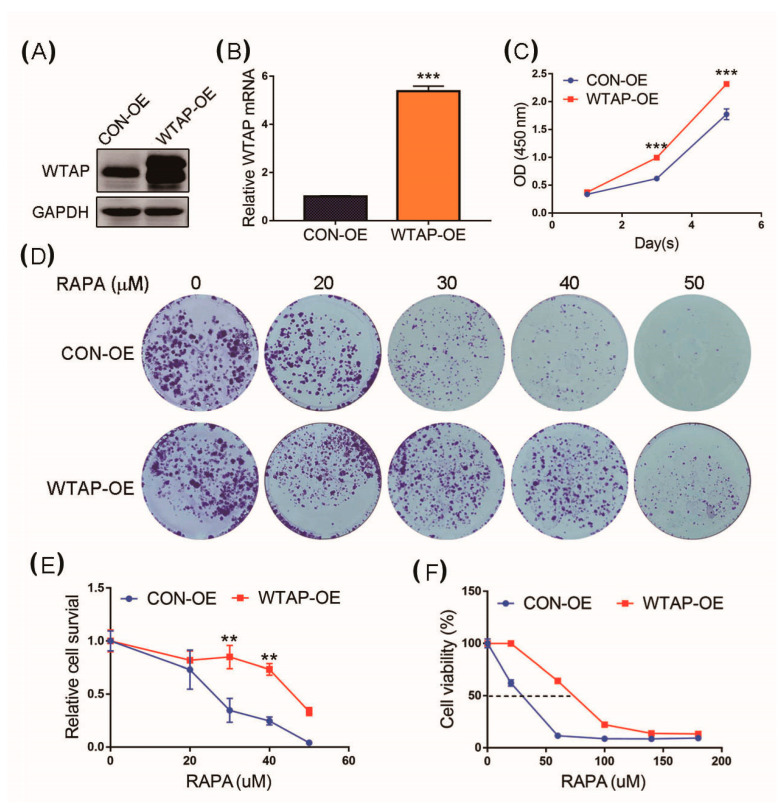
Effect of overexpression of WTAP on cell proliferation. (**A**) Western blot analysis of WTAP expressed in WTAP-OE and CON-OE cells. (**B**) RT-qPCR analysis of WTAP transcripts from WTAP-OE and CON-OE cells. (**C**) CCK-8 analysis was made to examine cell proliferation. (**D**) Colony formation analysis of WTAP-OE cells and CON-OE cells treated with different concentrations of RAPA. (**E**) Statistical analysis of cell survival of WTAP-OE cells and CON-OE treated with RAPA. (**F**) Statistical analysis of cell viability of WTAP-OE cells and CON-OE treated with RAPA. Error bars indicate the mean ± SD from three independent experiments (*n* = 3). ** indicate a statistically significant difference (*p* < 0.01), and *** indicates a statistically significant difference (*p* < 0.001).

A cell proliferation analysis was further used to study the effect of WTAP on the biological functions of HCC. A stable overexpression strain of WTAP fusion with EGFP in the HCC cell line SMMC-7721 was constructed through a lentiviral infection with subsequent puromycin screening. The overexpression of WTAP fusion with EGFP in the stable strain SMMC-7721 (labeled with SMMC-7721 WTAP-OE) and the control group (labeled with SMMC-7721 CON-OE) was used in the study. In the WTAP-OE lane of Figure 5A, the higher molecular weight band is the expression of WTAP fusion with EGFP, and the lower molecular band is the basal expression of WTAP of the HCC cell. Therefore, the Western blot results indicated that the expression level of WTAP in SMMC-7721 WTAP-OE was higher than that in SMMC-7721 CON-OE (Figure 5A). RT-qPCR results showed that the level of WTAP transcript in SMMC-7721 WTAP-OE was higher than that in SMMC-7721 CON-OE (Figure 5B). After the overexpression of WTAP was verified in SMMC-7721 WTAP-OE, the effect of the overexpression of WTAP on tumor cell proliferation was further evaluated by a CCK-8 assay. The SMMC-7721 CON-OE cells and SMMC-7721 WTAP-OE cells were seeded into 96-well plates, and a cell proliferation assay was created. The results showed that the proliferation ability of the SMMC-7721 WTAP-OE cells was better than that of the SMMC-7721 CON-OE cells (Figure 5C).

The knockdown of WTAP could facilitate the autophagy level of HCC. To further study whether WTAP affected cell proliferation by the regulation of autophagy, an autophagy activator rapamycin (RAPA) was used to perform the assay of colony formation. The results showed that the WTAP-OE cells had a stronger resistance to the inhibitory proliferation caused by RAPA-stimulated autophagy compared with that of CON-OE (Figure 5D). Furthermore, a statistical analysis showed that there was a significant difference in the cell survival between WTAP-OE and CON-OE (Figure 5E), as well as cell viability (Figure 5F). Therefore, the overexpression of WTAP resulted in the inhibition of autophagy, but it was beneficial to cell proliferation. 

**Figure 6:**
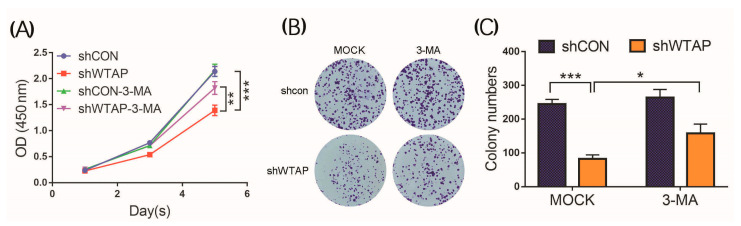
Effect of 3-MA on the proliferation of shWTAP-treated and shCON cells. (**A**) Determination of OD450 of shCON, shWTAP, shCON-3-MA, and shWTAP-3-MA cells at indicated times. (**B**) Colony formation analysis of shCON cells and shWTAP cells treated with 3-MA. (**C**) Statistical analysis of cell colonies between MOCK and 3-MA treatment groups. Error bars indicate the mean ± SD from three independent experiments (*n* = 3). *** indicate a statistically significant difference (*p* < 0.001). * indicate a statistically significant difference (*p* < 0.05) and ** indicate a statistically significant difference (*p* < 0.01).

3-MA is an inhibitor that can efficiently block the formation of autophagosomes. To further confirm the fact that shWTAP could facilitate autophagy in HCC, 3-MA (5 mM) was used to treat the cells for the analysis of cell proliferation. The results indicated that the number of shWTAP-treated cells was much lower than that of the shCON-treated cells. However, the number of shWTAP-treated cells was significantly increased after the treatment with 3-MA compared with that of the shWTAP group (Figure 6B). The OD_450_ value results also confirmed that the 3-MA treatment was facilitated to promote the growth of shWTAP-treated cells compared with that of shWTAP-treated Mock (Figure 6A). Furthermore, a statistical analysis (Figure 6C) showed that no significant difference in cell survival was found between the shCON-Mock and shCON group treated with 3-MA, but there was a significant difference in cell survival between the shWTAP-Mock and shWTAP group treated with 3-MA. Therefore, it was regarded that the knockdown of WTAP could facilitate the upregulation of autophagy in HCC.

**Figure 7:**
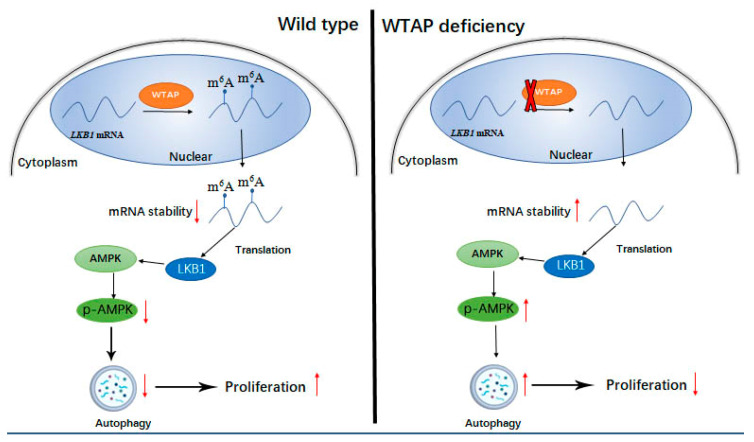
Schematic diagram for the regulatory role of WTAP on autophagy and proliferation of HCC, ↑ and ↓indicating upregulation and downregulation of target proteins, respectively. WTAP expression was not knocked down in wild type HCC. WTAP deficiency indicates the knockdown of WTAP in HCC by siRNA.

To demonstrate the effect of WTAP on autophagy, a model was established in Figure 7 to demonstrate the regulatory role of WTAP on the autophagy and proliferation of HCC. According to the mode, it was regarded that WTAP could increase the m^6^A modification of LKB1 mRNA and could diminish the stability of the LKB1 transcript, resulting in the decreased level of LKB1. Therefore, the decreased LKB1 could reduce the phosphorylation of AMPK, and the reduction in p-AMPK could impair the autophagy of HCC to some degree.

## 4. Discussion

Autophagy is an evolutionarily conserved intracellular degradation process through lysosomal hydrolases, and is regarded to play a dual role in cancers [27,28,29]. On the one hand, autophagy can induce the autophagic death of tumor cells and inhibit the growth of tumor cells. On the other hand, tumors can exploit autophagy to survive chemotherapeutic drug treatments. Therefore, autophagy is a promising target for drug development in cancer. Recently, m^6^A, an important epigenetic modification of RNA, has caused a growing worldwide concern, as it is reported to be associated with mRNA stability, translation, subcellular localization, and alternative splicing [16,17,30]. Currently, the accumulated evidence shows that m^6^A modification can regulate various of cellular biological processes including autophagy and metabolite synthesis [31,32]. However, the dysregulation of autophagy mediated by m^6^A may result in the occurrence of some diseases, even cancers. Therefore, it is very interesting to demonstrate the correlation between m^6^A and the autophagy of HCC in the study. This can provide not only a theoretical significance but also clinical treatment in tumors. 

ULK1, ATG5, and ATG7 are well-known autophagy-related proteins that participate in the formation of autophagosomes [33]. Studies reported that the transcripts of these proteins could be regulated by an m^6^A modification to modulate autophagy [22,23]. Studies further revealed that the *ATG5* and *ATG7* transcripts with a high m^6^A could be easily degraded, resulting in the reduction in target proteins. Furthermore, this also resulted in the reduction in autophagosomes and the inhibition of autophagy to some degree [23]. In this study, we found that the mechanism of the LKB1 transcript stability regulated by WTAP was similar to that of the *ATG5* and *ATG7* transcripts. The expression of WTAP could increase the m^6^A modified LKB1 but decreased the stability of the LKB1 transcript compared with that of the LKB1 mRNA from siWTAP-treated cells. Furthermore, the impaired stability of LKB1 caused by WTAP could reduce the expression level of LKB1, resulting in the decreased phosphorylation of AMPK and inhibition of autophagy. On the contrary, the knockdown of WTAP could decrease the m^6^A modified LKB1 but improve the stability of the LKB1 transcript, resulting in the increased expression of LKB1. Furthermore, this could promote the phosphorylation of AMPK and the increased level of p-AMPK could facilitate the autophagy level of HCC. Zhang et al. reported that LKB1 and AMPK could form a complex for AMPK activation [34]. In the study, our results further revealed that the phosphorylation of AMPK was upregulated through the increased level of LKB1 caused by the knockdown of WTAP, and a model shown in Figure 7 was established to illustrate the regulatory role of WTAP in autophagy. Another model was proposed by Chen et al.; that the m^6^A level of cellular transcripts in Leydig cells was decreased by both the downregulation of METTL14 and the upregulation of ALKBH5, and it could retard the m^6^A-mediated decay of cellular transcripts to upregulate CAMKK2 expression, resulting in the activation of PRKAA2 and autophagy initiation [32]. In contrast to the model proposed by Chen et al., the stability of m^6^A-mediated LKB1 transcripts of regulated HCC were decreased in the study. It was regarded that the difference in the m^6^A-mediated transcripts stability may be involved with the microenvironment of different cell lines and the structure of specific transcripts. Another possibility is that the stability of the m^6^A-mediated transcripts may be diversified in different transcripts. Therefore, further research will be required to study the stability of the transcripts mediated by m^6^A modification.

Additionally, we found that the AMPK signaling pathway participated in the regulation of autophagy through the phosphorylation of AMPK. AMPK is a key regulator of energy balance expressed ubiquitously in eukaryotic cells, and it is studied extensively in energy-sensing pathway [35]. To date, some reports have shown the correlation between the AMPK-dependent phosphorylation of Ulk1 and autophagy [36,37,38]. However, the biochemical details between AMPK and autophagy have not been fully elucidated. In the current study, we found that the knockdown of WTAP could facilitate the autophagy of HCC. To further demonstrate the mechanism by which WTAP-mediated m^6^A modification regulated autophagy, analyses of RNA-seq and m^6^A-seq were used to identify the downstream targets of WTAP-mediated m^6^A modification. LKB1, the upstream kinase of AMPK was found to be regulated by m^6^A, which could modulate cell autophagy at the molecular level.

## 5. Conclusions

In conclusion, we showed that the WTAP/LKB1/AMPK axis in HCC cells acted as a key regulator, linking m^6^A with autophagy. WTAP-mediated m^6^A modification plays an important role in the regulation of autophagy in HCC cells, which may provide a promising target for the treatment of HCC.

## Data Availability

The datasets used and/or analyzed during the current study are available from the corresponding author on reasonable request.
