# Correction: Li et al. m^6^A mRNA Methylation Regulates LKB1 to Promote Autophagy of Hepatoblastoma Cells through Upregulated Phosphorylation of AMPK. *Genes* 2021, *12*, 1747

**DOI:** 10.3390/genes14081575

**Published:** 2023-08-01

**Authors:** Guohui Li, Liang Deng, Nan Huang, Zhongqi Cui, Qi Wu, Ji Ma, Qiuhui Pan, Fenyong Sun

**Affiliations:** 1 School of Life Sciences, Jiangsu University, Zhenjiang 212013, China; ghli@ujs.edu.cn (G.L.); denglianghp@163.com (L.D.); 2 Department of Clinical Laboratory Medicine, Shanghai Tenth People’s Hospital of Tongji University, Shanghai 200072, China; huang_nan2021@163.com (N.H.); 18817527896@163.com (Z.C.); wuqi496@163.com (Q.W.); 3 Department of Laboratory Medicine, Shanghai Children’s Medical Center, Shanghai Jiao Tong University School of Medicine, Shanghai 200072, China; maji@scmc.com.cn (J.M.); panqiuhui_med@163.com (Q.P.); 4 Shanghai Key Laboratory of Clinical Molecular Diagnostics for Pediatrics, Shanghai 200072, China

## Error in Figure

In the original publication [1], there was a mistake in Figure 1G. In the shCon panels of Figure 1G, it was our carelessness to make the cells shift left during the processing of images of the cells fixed with DAPI. So, the images in the shCON panels of Figure 1G did not correctly overlay with the other panels, and some readers have pointed out the mistake to us. The corrected Figure 1 is presented below.

The authors state that the scientific conclusions are unaffected. This correction was approved by the Academic Editor. The original publication has also been updated.

## Figures and Tables

**Figure 1:**
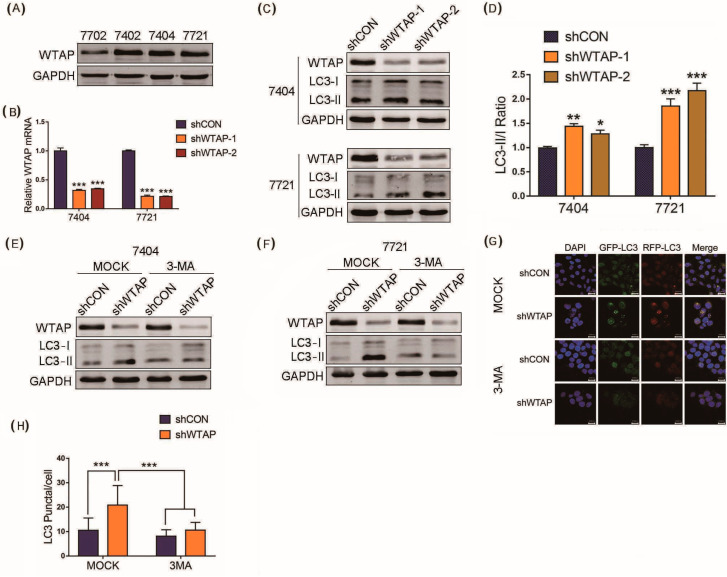
Knockdown of WTAP facilitating autophagy of HCC. (**A**) Western blot analysis of basal expression of WTAP in different HCC cell lines. (**B**) RT-qPCR analysis of WTAP transcripts from shWTAP cells and shCON cells. (**C**) Western blot analysis of LC3-I and LC3-II in shWTAP SMMC-7721 cells and shWTAP BEL-7404 cells. (**D**) Statistical analysis of LC3 II/LC3 I ratio between shCON and shWTAP group. (**E**) Western blot analysis of LC3-I and LC3-II in WTAP-knockdown BEL-7404 cells treated with or without 3-MA. (**F**) Western blot analysis of LC3-I and LC3-II in WTAP-knockdown SMMC-7721 cells treated with or without 3-MA. (**G**) Cofocal microscopy analysis of LC3 in shWTAP cells treated with or without 3-MA. (**H**) Statistical analysis of LC3 puncta in shWTAP cells and shCON cells, as well as 3-MA-treated cells. Error bars indicate the mean ± SD from three independent experiments (*n* = 3). *p* values was used to indicate statistica difference. * *p* < 0.05, ** *p* < 0.01 and *** *p* < 0.001.
